# The Complexome of *Dehalococcoides mccartyi* Reveals Its Organohalide Respiration-Complex Is Modular

**DOI:** 10.3389/fmicb.2018.01130

**Published:** 2018-06-12

**Authors:** Katja Seidel, Joana Kühnert, Lorenz Adrian

**Affiliations:** ^1^Department Isotope Biogeochemistry, Helmholtz Centre for Environmental Research–UFZ, Leipzig, Germany; ^2^Chair of Geobiotechnology, Technische Universität Berlin, Berlin, Germany

**Keywords:** *Dehalococcoides*, organohalide respiration, anaerobic respiration, reductive dehalogenase, protein complex, complexome analysis, blue native gel electrophoresis

## Abstract

*Dehalococcoides mccartyi* strain CBDB1 is a slow growing strictly anaerobic microorganism dependent on halogenated compounds as terminal electron acceptor for anaerobic respiration. Indications have been described that the membrane-bound proteinaceous organohalide respiration complex of strain CBDB1 is functional without quinone-mediated electron transfer. We here study this multi-subunit protein complex in depth in regard to participating protein subunits and interactions between the subunits using blue native gel electrophoresis coupled to mass spectrometric label-free protein quantification. Applying three different solubilization modes to detach the respiration complex from the membrane we describe different solubilization snapshots of the organohalide respiration complex. The results demonstrate the existence of a two-subunit hydrogenase module loosely binding to the rest of the complex, tight binding of the subunit HupX to OmeA and OmeB, predicted to be the two subunits of a molybdopterin-binding redox subcomplex, to form a second module, and the presence of two distinct reductive dehalogenase module variants with different sizes. In our data we obtained biochemical evidence for the specificity between a reductive dehalogenase RdhA (CbdbA80) and its membrane anchor protein RdhB (CbdbB3). We also observed weak interactions between the reductive dehalogenase and the hydrogenase module suggesting a not yet recognized contact surface between these two modules. Especially an interaction between the two integral membrane subunits OmeB and RdhB seems to promote the integrity of the complex. With the different solubilization strengths we observe successive disintegration of the complex into its subunits. The observed architecture would allow the association of different reductive dehalogenase modules RdhA/RdhB with the other two protein complex modules when the strain is growing on different electron acceptors. In the search for other respiratory complexes in strain CBDB1 the remarkable result is not the detection of a standard ATPase but the absence of any other abundant membrane complex although an 11-subunit version of complex I (Nuo) is encoded in the genome.

## Introduction

**Figure 1:**
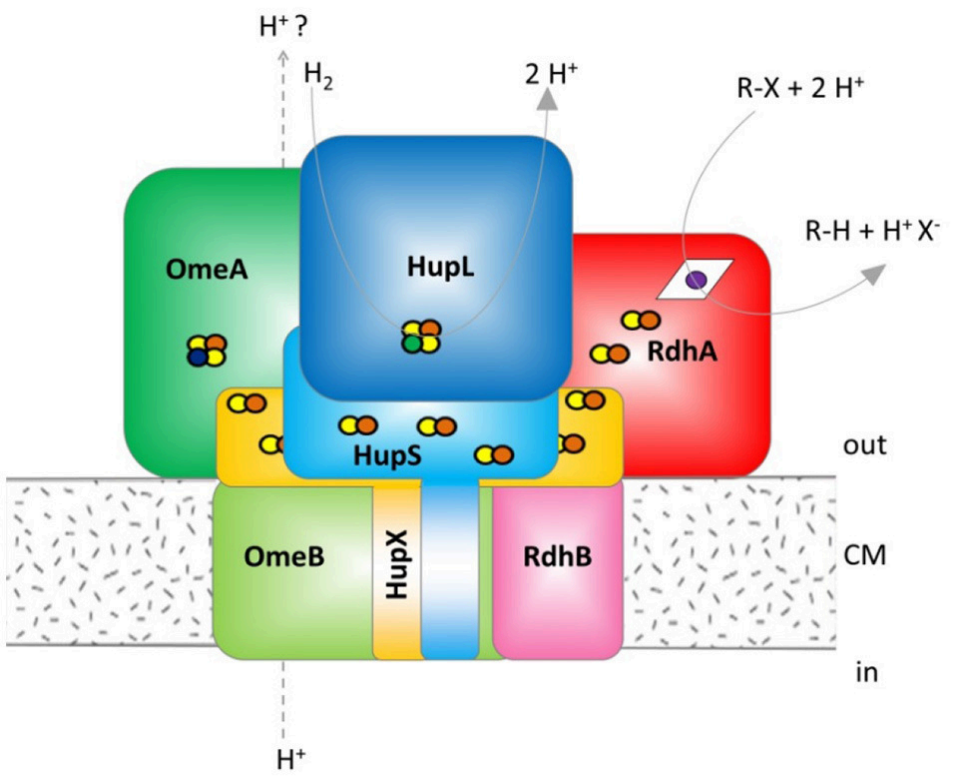
Hypothetical model of the organohalide complex, modified from Kublik et al. (2016) representing multiple interactions between subunits. HupL, hydrogenase large subunit 48 kDa; HupS, hydrogenase small subunit 37.1 kDa, HupX, iron/sulfur protein 30.4 kDa, OmeA, organohalide respiration involved molybdoenzyme A 106 kDa; OmeB, organohalide respiration involved molybdoenzyme B 44.8 kDa; RdhA, reductive dehalogenase homologous protein active subunit ~55 kDa; RdhB, reductive dehalogenase homologous anchoring protein B 11.2 kDa. The colors were defined (Supplemental Table 2) and used throughout the manuscript for all graphs and tables. The yellow/red dots indicate Fe/S clusters, the blue dot indicates a molybdenum ion, the green dot a nickel ion and the violet dot a cobalt ion bound to a corrinoid.

Organohalides are used in many different products such as pesticides, biocides, pharmaceuticals, plasticizers, personal care articles and flame retardants but are also produced by natural processes. Released into the environment they often represent persistent, toxic and bioaccumulating legacy contaminants (Jones and de Voogt, 1999). One particular process favored for bioremediation of organohalides in sediments, soils or groundwater is organohalide respiration catalyzed by organohalide-respiring bacteria (Steffan and Schaefer, 2016). Organohalide-respiring bacteria use the halogenated compounds as a terminal electron acceptor in an anaerobic respiration and gain energy for growth from this process. Several organohalide-respiring bacteria are strictly anaerobic and obligate organohalide respiring including strains of the genera *Dehalococcoides, Dehalogenimonas* and *Dehalobacter* separating them from many other described organohalide-respiring bacteria (Jugder et al., 2016; Fincker and Spormann, 2017). *Dehalococcoides mccartyi* strain CBDB1 is physiologically well characterized and its genome is sequenced (Kube et al., 2005). The strain can grow with hydrogen as sole electron donor and various halogenated electron acceptors, including chlorinated and brominated benzenes, polychlorinated dioxins, polychlorinated biphenyls, chlorophenols, oligocyclic phenolic bromoaromatics, and perchloroethene (Bunge et al., 2003; Adrian et al., 2009; Yang et al., 2015). The key enzyme in reductive dehalogenation is the reductive dehalogenase (RdhA), a 50–60 kDa protein with a cobalamin and two iron-sulfur clusters as co-factors. A Tat-leader peptide indicates its export to the outer side of the monoderm cell envelop of *Dehalococcoides* (Schubert et al., 2018). Earlier, we have obtained evidence that the RdhA protein is coordinated in a multi-protein reductive dehalogenase complex, referred to as organohalide respiration complex (OHR complex) in the text. The current hypothesis is that the OHR complex represents a fully functional stand-alone respiratory chain, obviating quinone or cytochrome involvement (Kublik et al., 2016; Hartwig et al., 2017). The other proposed subunits in the OHR complex are RdhB, which is the putative membrane integral RdhA anchoring protein, the *o*rganohalide respiration involved *m*olybdo*e*nzyme (OmeA), its putative membrane-integrated anchor OmeB, HupX, a protein with four predicted iron-sulfur clusters and a hydrogen uptake hydrogenase with its [NiFe] large subunit (HupL) and iron-sulfur containing small subunit (HupS) (Figure 1). RdhB and OmeB are predicted to be membrane integrated, while HupX and HupS are predicted to be membrane attached via one transmembrane domain each. The current mechanistic model hypothesizes that electrons are fed into the complex via HupL, and then transferred via iron-sulfur clusters in HupS, HupX and OmeA to the RdhA which reduces organohalides. The electric current through this electron chain might induce conformational changes in the complex that drive proton translocation across the membrane, but nothing of this is yet tested. The reaction would in some way resemble the reaction in the Rnf complex, where a redox reaction drives the translocation of cations across the membrane in an autonomous protein complex. The Rnf complex uses the redox reaction between reduced ferredoxin and NAD^+^, both located in the cytoplasm, to shuttle Na^+^ ions (Imkamp et al., 2007; Hess et al., 2013). In contrast the OHR complex is believed to take both redox substrates from outside the cell. The role of the molybdopterin dinucleotide binding motif in OmeA is not understood and the formation of such a molybdenum-containing cluster is not confirmed. The genes for the seven different OHR complex proteins are distributed into different operons on the genome. The genes *hupL, hupS*, and *hupX* are co-localized while *omeA* and *omeB* are located together on a separate locus. The reductive dehalogenases constitute a large paralog group in *D. mccartyi* strain CBDB1 with 32 different loci on the genome each containing one *rdhA* and one *rdhB* gene plus regulatory genes (Schubert et al., 2018).

Evidence for the formation of a dehalogenating multiprotein complex came from experiments with blue native gels where biochemical activity was detected in gel slices corresponding to a molecular weight of 150–250 kDa, but not in gel areas corresponding to the molecular weight of a single RdhA subunit (Kublik et al., 2016). In addition activity was detected at high molecular weight fractions in gel filtration experiments and could also not be found in the eluate after ultrafiltration at 100 kDa exclusion pore size. Subsequently, the mass spectrometric analysis of bands in blue native gels gave evidence for the composition of the OHR complex. No experimental evidence however, is available yet for the interaction of single proteins within the complex and it is not clear which proteins mediate the cohesion of the complex. In addition no experimental evidence is yet available showing a specific interaction of an RdhA with its genome co-localized RdhB. The two most abundant RdhA proteins in cultures of strain CBDB1 growing with chlorobenzenes as electron acceptor are the RdhA proteins with the locus tags CbdbA80 and CbdbA84. CbdbA84 was isolated from a native gel and showed dechlorination activity with chlorinated benzenes. Accordingly it was annotated as chlorobenzene reductase (CbrA) (Adrian et al., 2007b). The experimental work with *Dehalococcoides* strains is strongly affected by the extremely low amounts of cell mass obtained from cultivation. The dependence of growth on toxic, low-soluble electron acceptors as well as the slow growth rate of the organisms of about 3 days per division results in maximum cell numbers of about 1–5 × 10^8^ cells per mL in a fully grown culture. This, however, represents only about 1–5 mg protein per liter in cultures because the cells are very small (0.5 × 0.5 × 0.2 μM) and contain low amounts of protein compared to other microorganisms usually cultivated in the laboratory. The lack of cell walls and the small size also reduces harvesting efficiency. Therefore, all biochemical experiments with *Dehalococcoides* strains are performed with protein amounts below 50 μg total protein and all biochemical experimental approaches are restricted to highly sensitive methods such as activity tests, gel electrophoresis and mass spectrometry. Whereas, a cloning system is not available for *Dehalococcoides* species due to low growth rates, first progress was recently achieved with heterologous expression of *Dehalococcoides* redox proteins in *E. coli* (Hartwig et al., 2015).

Complexome analysis (also called complexome profiling) is an approach in which protein complexes are mildly extracted from cells and applied to non-destructive methods to separate protein complexes from each other. Density gradient centrifugation, gel filtration and native gel electrophoresis have been applied for protein separation but only native gel electrophoresis allows very sensitive detection when a low amount of material is available (Heide et al., 2012). To exclude bias from personal hypotheses, the entire gel lane can be cut into small slices and each slice is analyzed for protein content by mass spectrometry. Mass spectrometry gives a great depth of detection and estimates of the quantitative distribution of detected proteins across the gel lane. By identifying correlating distribution patterns, evidence for protein-protein interactions and the formation of protein complexes can be obtained. The method was described before for mitochondrial complexes in mammals (Wittig et al., 2006; Heide et al., 2012), plants (Kiirika et al., 2013; Senkler et al., 2017), and yeast (Eydt et al., 2017), and bacterial protein complexes of sulfate-reducing (Wöhlbrand et al., 2016), oxygen respiration in nitrate-reducing (Schimo et al., 2017) and anammox bacteria (de Almeida et al., 2016). A complication of complexome analyses is the fact that many of the protein complexes, especially those involved in respiration, are membrane bound and detergents must be applied to detach protein complexes from the membrane. The solubilization step with detergents, however, is crucial as solubilization needs to be strong enough to extract membrane-integrated proteins but weak enough to keep protein 3-D structures and protein-protein interactions intact.

The objective of this study is to obtain more and clearer evidence on the organization of the unique quinone-free respiratory complex in *D. mccartyi* strain CBDB1. Due to the low amount of biomass available we were restricted to methods applicable with low amounts of protein. Therefore, we performed a focused complexome analysis with the main aim to analyse protein components and modules of the OHR complex rather than measuring all protein-protein interactions in the proteome. This approach based on earlier data showed that the proteins in the OHR complex are all at high relative abundance compared to anabolic or structural proteins in the strain. The application of low protein amounts onto blue native gels therefore excluded the detection of low-abundance complexes and proteins but was expected to improve the resolution on the gel. To obtain information on the different strengths of protein-protein interactions we applied three different protocols for protein extraction from cells varying in their solubilization strength.

## Materials and methods

### Chemicals, cultures, and cell harvest

Chemicals were purchased at the highest available purity. Hexachlorobenzene of technical purity was purchased from Campro Scientific and 1,2,4,5-tetrabromobenzene from Alfa Aesar at a purity of 94%. Titanium(III) citrate was prepared from technical grade 15% titanium(III) chloride solution (Merck-Schuchard) as described (Adrian et al., 2007a).

*Dehalococcoides mccartyi* strain CBDB1 was grown in defined mineral medium, pH-buffered to 7.2 with carbonate, reduced with 2 mM L-cysteine or 0.15% titanium(III)citrate, with 5 mM acetate as carbon source, 7.5 mM nominal concentration of hydrogen as electron donor and hexachlorobenzene or 1,2,4,5- tetrabromobenzene as electron acceptor under strictly anaerobic conditions as described previously (Adrian et al., 2000). Cultivation led to a final cell density of about 10^7^-10^8^ cells mL^−1^. Cells were harvested from culture volumes of 400–700 mL by using a SandTrap as recently described (Frauenstein et al., 2017). Eluents from the SandTrap were subsequently concentrated by a multi-step centrifugation (2–6 steps) each at 6,000 × g and 16°C for 60 min, removing about 50% of the supernatant in each step to avoid the strong losses of cells by other procedures as described in detail previously (Frauenstein et al., 2017). Due to low cell numbers harvested and the small cell size of the bacteria no visible pellet was formed. Nevertheless, as calculated from monitoring the centrifugation steps by direct epifluorescence microscopic cell counting after SYBR-green staining on agarose-coated slides (Adrian et al., 2007a) this non-visible pellet containing about 10^9^ cells was used for further steps. Assuming a protein content of 30 fg protein cell^−1^ (Cooper et al., 2015) this represents about 30 μg protein as starting material for each of the described native electrophoresis/complexome analyses. The details for each of the three complexome analyses described in this study are summarized in Table 1.

**Table 1:** Experimental details in complexome analyses with the three different extraction modes.

**Experimental stage**	**Experimental parameter**	**Complexome analysis 1**	**Complexome analysis 2**	**Complexome analysis 3**
Culture	Culture name	LB99c	LB104c	LB99b
	e^−^-Acceptor	HCB	1,2,4,5-TeBB	HCB
	Reducing agent	L-Cysteine	L-Cysteine	Titanium(III)citrate
	Culture volume (mL)	700	400	650
	Culture cell number (cells mL^−1^)	2.7E+07	7.0E+07	5.7E+07
SandTrap harvest	SandTrap size	SandTrap GL32	SandTrap GL25	SandTrap GL32
	Volume after SandTrap	25 mL	9 mL	30 mL
	Cell number after SandTrap (cells mL^−1^)	1.3E+08	7.0E+08	1.3E+08
Centrifugation steps	Centrifugation condition	6x centrifugation each 6,000 *g*, 1 h, 16°C; ~50% of the SN removed	4x centrifugation each 6,000 *g*, 1 h, 16°C; ~50% of the SN removed	2x centrifugation each 6,000 *g*, 1 h, 16°C; ~50% of the SN removed
	Volume after concentration (mL)	0.1	0.25	0.85
	Cell number after concentration (cells mL^−1^)	1.4E+09	3.1E+10	3.0E+09
Solubilization	Extraction mode	1	2	3
		“whole-cell DDM extraction”	“membrane fraction DDM extraction”	“disrupted cell DDM extraction”
	Extraction procedure	solubilization	beat-beating then ultracentrifugation then solubilization	beat-beating and solubilization
		***During solubilization an unknown amount of cells/protein is lost***		
BN-PAGE	Total volume added to each lane (μL)	35	25	35
	Volume of cell extract added per lane (μL)	25	18.125	25
Calculated amounts if no loss occurred during extraction	Cells per lane	3.5E+07	5.6E+08	7.5E+07
	Protein per lane (μg)	1.1	16.9	2.3
Mass spectrometry	Measured total area counts of detected proteins^a^	3.9E+10	1.9E+10	1.8E+10

a *see also Table 2*.

### Extraction of protein complexes

Proteins were extracted from the non-visible pellet in three different ways with the aim to detach complexes from the membrane while preserving protein-protein interactions as much as possible.

- In the first extraction mode, previously used in our group (Kublik et al., 2016), the cell pellet was resuspended in 1 × PBS buffer (10 mM disodium hydrogen phosphate, 2 mM potassium dihydrogen phosphate, 137 mM sodium chloride, 2.7 mM potassium chloride, pH 7.4) and amended with 1% (w/v) N-dodecyl-ß-D-maltoside (DDM). The suspension was incubated for 60 min on ice with gentle shaking. Insoluble material was then removed by centrifugation (45 min, 16,600 × g, 16°C) and the supernatant was used for blue native polyacrylamide gel electrophoresis (BN-PAGE). We refer to this extraction mode as “whole-cell DDM extraction.”
- In the second extraction mode, previously described for sulfate reducing bacteria (Wöhlbrand et al., 2016), the pellet was suspended in 250 μL culture medium and cells were lysed by bead beating (0.5 mm glass beads, Stretton Scientific; Fast Prep TM FP120, Thermo Savant) at a speed of 4 m s^−1^ for 20 s at room temperature. Beads were removed by centrifugation (3,000 × g, 1 min, room temperature) and the supernatant was processed by ultracentrifugation (100,000 × g, 60 min, 4°C) to spin down membrane fractions. The ultracentrifugation supernatant was removed and the pellet frozen overnight at −80°C under anoxic conditions. Afterwards the membrane pellet was gently thawed and resuspended in ACA750 buffer (Wöhlbrand et al., 2016) (750 mM amino capronic acid, 50 mM Bis-Tris, 0.5 mM EDTA, pH 7.0) containing 1% (w/v) DDM. Solubilization was performed by gently shaking for 60 min at 4°C. This extraction mode will be referred as “membrane fraction DDM extraction.”
- For the third extraction mode the cell pellet was resuspended in 850 μL 1 × PBS containing 1% (w/v) DDM (at room temperature) and the cells were then disrupted by bead beating applying 4 m s^−1^ for 30 s at room temperature. This resulted in the formation of a minor amount of foam. The resulting crude extract was then centrifuged for 15 min at 14,000 × g and 4°C. The supernatant with solubilized proteins was transferred to a fresh tube and stored on ice for several minutes before the sample was loaded on blue native polyacrylamide gel. This extraction mode will be referred to as “disrupted cell DDM extraction.”

### BN-PAGE

BN-PAGE was performed in an anoxic glove box and cooled with cooling packs using the XCell SureLock Mini-Cell (Invitrogen) electrophoresis system with precast 4–16% gradient Bis-Tris gels (NativePAGE Novex, Invitrogen). The anode buffer and the light blue cathode buffer were prepared according to the manufacturer's manual. A protein marker (5 μL of 1:20 diluted NativeMark Unstained Protein Standard, Invitrogen) in 1 × sample buffer (Invitrogen) was used in one lane of all gels. Samples were amended with 4 × sample buffer and 0.125–0.178% (v/v) Coomassie G-250 according to the manufacturer's instructions before electrophoresis was run at 150 V for 1 h and then at 250 V until the marker reached the bottom of the gel. After electrophoresis proteins were visualized by a mass spectrometry-compatible silver staining procedure (Nesterenko et al., 1994). Then the stained gel lanes were cut into regular slices of 1–2 mm width using a razor blade that was cleaned with ethanol after each cut. In the three different complexome analyses the gels were cut into 26, 65 and 64 slices, respectively.

### Mass spectrometric analysis

Silver stained proteins in gel slices were prepared for nano-liquid chromatography tandem mass spectrometry (nLC-MS/MS) analysis by destaining, cysteine derivatization and tryptic in-gel digest as described (Kublik et al., 2016). Peptide samples were desalted using ZipTip-μC18 material (Merck Millipore) prior to analysis with nLC-MS/MS using an Orbitrap Fusion Tribrid mass spectrometer (Thermo Scientific) equipped with a nanoLC system (Dionex Ultimate 3000RSLC; Thermo Scientific). Peptides were separated on a separation column (Acclaim PepMap100 C18, Thermo Scientific) at a flow rate of 0.3 μL min^−1^ by applying the following settings with eluent A (0.1% formic acid in water) and eluent B (80% (v/v) acetonitrile, 0.08% formic acid in water): column equilibration for 3 min at 4% B (corresponding to 3.2% (v/v) acetonitrile final concentration), then increasing within 40 min to 55% B (corresponding to 44% (v/v) acetonitrile), followed by increasing within 1 min to 90% B (corresponding to 72% (v/v) acetonitrile) and hold for 4 min at 90% B. Eluted peptides were ionized via an electrospray ion source (TriVersa NanoMate, Advion) operated in positive mode and scanned continuously between 350 and 2,000 *m/z* by the Orbitrap mass analyzer with a resolution of 120,000, an ion target value of 4 × 10^5^ ions and maximum ion injection time of 50 ms. The two most intense ions with charge between 2+ and 7+ were picked for fragmentation with the quadrupole set to a window of 1.6 *m/z* and subjected either to higher energy collisional dissociation (HCD) mode with collision energy of 30% or collision induced dissociation (CID) mode with collision energy of 35% with an ion target value of 1 × 10^4^ and maximum ion injection time of 120 ms. Fragment ions were analyzed in the ion trap mass analyzer. Dynamic exclusion was enabled for 45 s after fragmenting a peptide ion in order to prevent repeated fragment analysis of the same ion.

Protein identification was conducted by Proteome Discoverer (v2.2, Thermo Fisher Scientific) using the SequestHT search engine with the UniProt database of *D. mccartyi* strain CBDB1 with the following settings: cleavage enzyme trypsin, allowing up to two missed cleavages, precursor mass tolerance and fragment mass tolerance were set to 3 ppm and 0.6 Da, respectively. Oxidation of methionine residues was selected as dynamic modification and carbamidomethylation on cysteine residues as fixed modification. The false discovery rate of identified peptide sequences was kept to <1% using the Percolator node. Abundance of proteins and peptides was calculated by label-free quantification on the basis of area counts using the Minora node implemented in Proteome Discoverer. The relative protein abundance or relative peptide abundance as given in this study was defined as the abundance of a protein or peptide in one slice relative to the total abundance of this protein or peptide across all slices of the blue native gel lane. The hierarchical cluster analyses were performed with R.

## Results

Three different extraction modes were applied (denominated as complexome analysis 1, 2, and 3) to release protein subcomplexes from the membrane of *D. mccartyi* strain CBDB1. The three extraction modes were designed to exert different solubilization strengths and therefore to provide different snapshots of subcomplex states in different dissociation degrees allowing conclusions on the stability of membrane-bound subcomplexes. Solubilized protein complexes were separated under non-denaturing conditions by BN-PAGE. Instead of cutting single bands after staining, the entire gel lane was cut into regular 1–2 mm slices and proteins within each slice were trypsin digested, eluted and analyzed by mass spectrometry. The result was an in-depth and high-resolution picture of protein distribution across the gel lane at three different solubilization degrees.

### Protein-protein interactions in the OHR complex after whole-cell DDM extraction (extraction mode 1)

**Figure 2:**
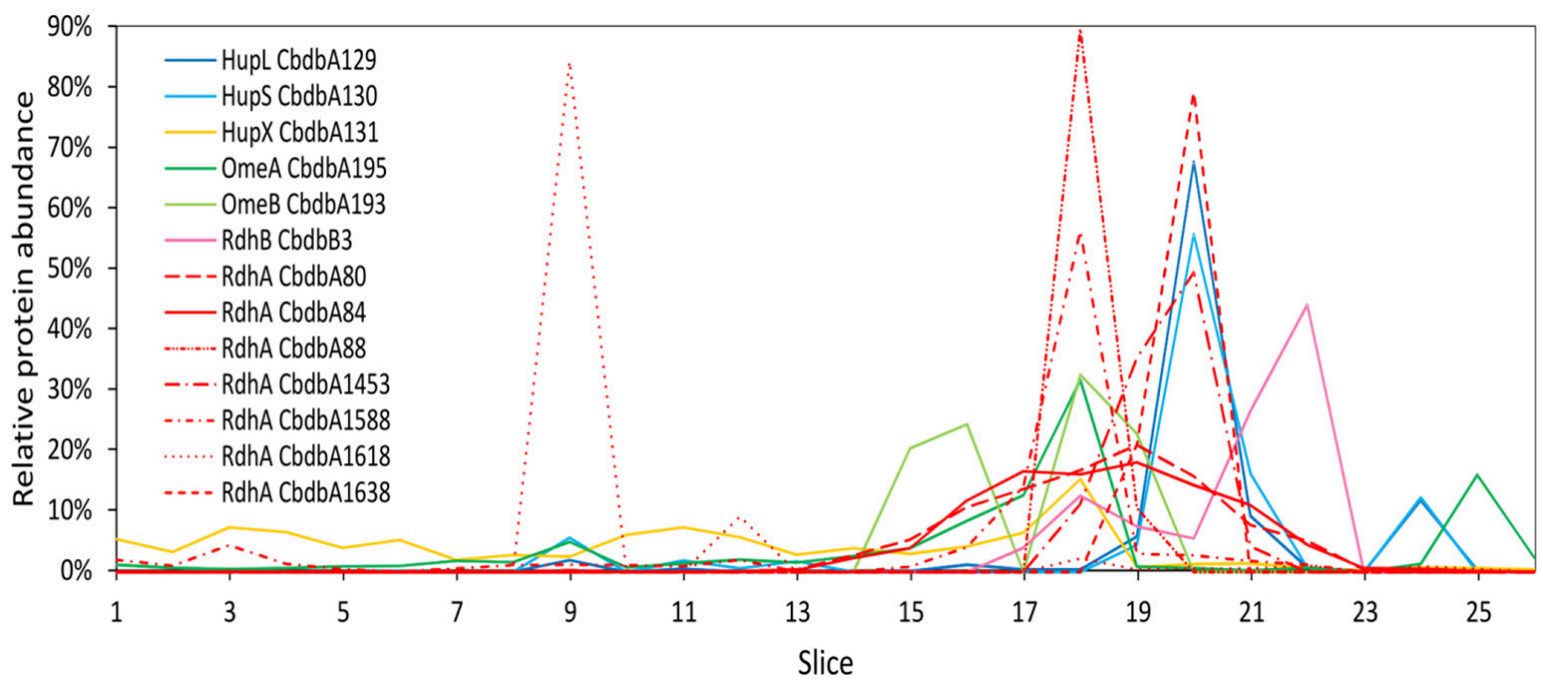
Relative abundance of proteins associated with the OHR complex across the length of a blue native gel cut into 26 slices after whole-cell DDM solubilization (protein extraction mode 1). The color code is the same as shown in Figure 1. Different RdhA proteins are shown with different line types.

Extraction mode 1, in which whole cells were incubated directly 60 min at 4°C with 1% (w/v) DDM, was the fastest and most direct protein extraction protocol tested in this study. This protocol was not designed to separate soluble cytoplasmic from membrane proteins but for having the fewest experimental steps avoiding protein loss. From the gel that was cut into 26 slices a total of 103 proteins were identified by mass spectrometry across all slices (Supplemental Table 1). All putative subunits of the OHR complex were identified: HupL, HupS, HupX, OmeA, OmeB, seven RdhA paralogs and one RdhB (Table 2). The distribution of the relative protein abundance of the OHR complex subunits across the gel lane showed that the OHR complex is organized in modules of several subunits under extraction mode 1 (Figure 2). The subunits HupL and HupS showed identical migration patterns and shared their maximum of relative protein abundance in slice 20, indicating that they were tightly connected to each other and that they were forming a hydrogenase module. Although OmeA and OmeB shared their maximum in slice 18, their overall relative protein abundance distribution pattern was not identical, as OmeB formed a second slightly smaller maximum of relative protein abundance in slice 16, which was not present for OmeA. In contrast to HupL and HupS, HupX had its maximum of relative protein abundance in slice 18, identical to OmeA and OmeB indicating that these three subunits OmeA, OmeB, and HupX also formed a tightly bound module of the OHR complex and that HupX was not tightly bound to HupS/HupL. Overall, the HupL/HupS module had a completely different migration pattern than the OmeA/OmeB/HupX module indicating that no strong interactions between the two modules were present. Seven out of 32 different RdhA proteins encoded in the genome were found in the gel. From the seven identified RdhA proteins five showed a clear maximum of relative protein abundance in one of the slices (CbdbA1618 in slice 9; CbdbA88 and CbdbA1588 in slice 18; CbdbA1453 and CbdbA1638 in slice 20). Our data might indicate that CbdbA88 and CbdbA1588 are more strongly attached to the OmeA/OmeB/HupX module whereas CbdbA1453 and CbdbA1638 are more strongly attached to the HupL/HupS module. In contrast to the five RdhA proteins with clear relative maxima, CbdbA80 and CbdbA84 were more broadly distributed between the slices 16 to 21, therefore covering the whole range of the other OHR complex proteins and no separation was achieved. Indeed CbdbA80 and CbdbA84 were by far the most abundant among all RdhA proteins (Table 2), indicating that a better resolution could be achieved with lower amounts of protein. Only one RdhB protein was identified with a single peptide. This RdhB protein with the locus tag CbdbB3 is encoded in one operon together with the RdhA CbdbA80 and might be the specific membrane anchor for CbdbA80. In the blue native gel CbdbB3 had its maximum in slice 22, where none of the other OHR complex subunits had a maximum. Therefore, most of this RdhB protein appeared to have been detached from the overall protein complex by extraction mode 1. However, a second smaller maximum in relative protein abundance of CbdbB3 showed up in slice 18 where several RdhA proteins were also detected, including RdhA CbdbA80.

**Table 2:** Absolute abundance values for all suspected OHR complex subunits as a sum of the determined mass spectrometric area counts in all slices.

**Protein**	**Extraction mode 1**	**Extraction mode 2**	**Extraction mode 3**
**Total detected proteins**	**103**	**233**	**116**
**Sum of all detected proteins**	**3.9E**+**10**	**1.9E**+**10**	**1.8E**+**10**
HupL CbdbA129	2.8E+08	2.7E+07	8.9E+07
HupS CbdbA130	8.1E+07	2.5E+06	2.1E+07
HupX CbdbA131	6.9E+08	4.6E+08	2.9E+07
OmeA CbdbA195	5.2E+08	7.7E+07	3.2E+08
OmeB CbdbA193	4.6E+06	7.3E+07	4.2E+07
RdhB CbdbB3	2.6E+07	1.0E+08	1.5E+08
RdhA CbdbA80	8.6E+08	9.0E+07	1.9E+09
RdhA CbdbA84	7.4E+09	9.4E+06	4.5E+09
RdhA CbdbA88	3.6E+06		
RdhA CbdbA1453	7.0E+06		
RdhA CbdbA1455	3.5E+05		
RdhA CbdbA1588	5.4E+08		7.4E+08
RdhA CbdbA1618	2.4E+08	3.3E+07	6.9E+06
RdhA CbdbA1638	1.8E+06		2.5E+06

*The color code for proteins is the same as elsewhere in this study. Protein abundance values are highlighted by gray shading corresponding to abundance values in each extraction mode (highly abundant, gray; lowly abundant, white)*.

### Protein-protein interactions in the OHR complex after membrane protein DDM extraction (extraction mode 2)

**Figure 3:**
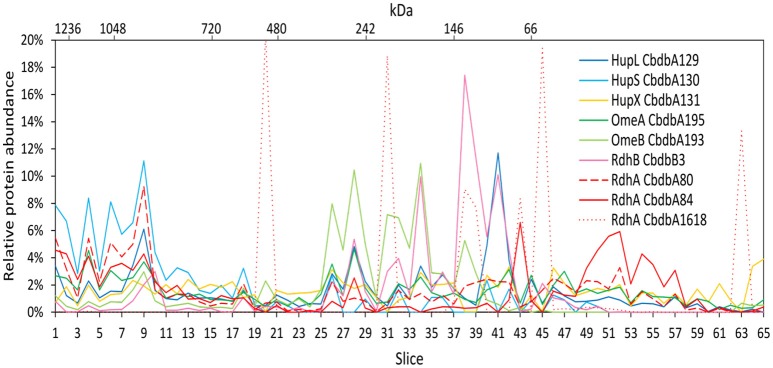
Relative abundance of proteins associated with the OHR complex across the length of a blue native gel cut into 65 slices after membrane fraction DDM extraction (protein extraction mode 2). The color code is the same as used in Figure 1. Different RdhA proteins are shown with different line types.

Extraction mode 2 was adapted from a previous complexome analysis of the sulfate reducing *Desulfobacula toluolica* (Wöhlbrand et al., 2016). The approach aimed at first isolating membrane fractions from which protein complexes were subsequently detached with DDM. The blue native gel lane was cut into 65 slices and 233 proteins were identified in total by mass spectrometry from all slices together (Table 2). Again all subunits of the complex were identified, but the distribution of the relative protein abundance across the gel lane appeared random instead of showing module formation (Figure 3). Overall only very few proteins had a relative abundance of more than 10% in one single slice and the total amount was often distributed across all 65 slices. One example was HupX which reached abundance of 1–3% in almost all 65 slices, but nowhere more than 5%. One exception was the RdhA CbdbA1618, which had clear maxima but was expressed at a very low absolute abundance (Table 2). More meaningful exceptions were the membrane integrated subunits HupS, OmeB and RdhB CbdbB3 which had local accumulations in the gel in the slices 1–9, 26–38, and 26–41 respectively. OmeB and RdhB CbdbB3 appeared to be co-localized suggesting strong interaction. HupS in contrast did clearly not interact with HupL as seen with extraction mode 1 and did not interact with the other two membrane subunits of the complex OmeB and RdhB but co-localized with RdhA CbdbA80. HupL had a high maximum of relative protein abundance in slice 41 at a size of ~100 kDa, which is not fitting to the predicted monomeric molecular mass of 58 kDa. In the blue native gel after extraction mode 2 we obtained only about half the total area counts across all proteins and slices than those obtained after extraction mode 1 (Table 2 and Supplemental Table 1). In contrast to this, more than twice as many proteins were detected in complexome analysis 2 compared to complexome analysis 1 (233 vs. 103). The extraction mode was aiming at enriching membrane proteins but also soluble proteins were present and enriched. Especially, many of the ribosomal proteins were detected with extraction mode 2 (33 identified ribosomal proteins in comparison to 2 ribosomal proteins detected under extraction modes 1 and 3) suggesting that ribosomes were enriching in the ultracentrifugation step. A similar effect was seen with the ATPase subunits of which five were detected after extraction mode 2 but only one after extraction mode 1 and three after extraction mode 3.

### Protein-protein interactions in the OHR complex after disrupted cells DDM extraction (extraction mode 3)

**Figure 4:**
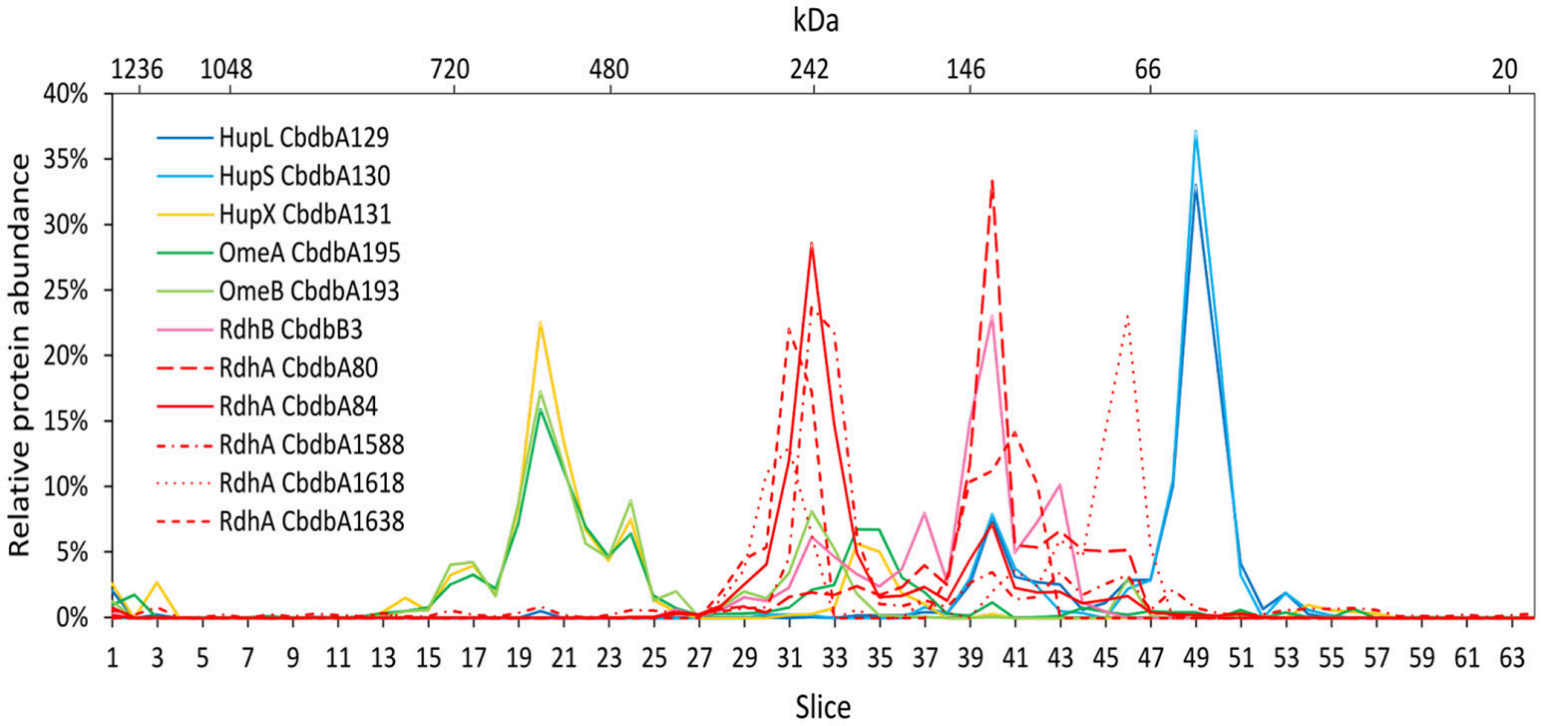
Relative abundance of proteins associated with the OHR complex across the length of a blue native gel cut into 64 slices after disrupted cell DDM extraction (protein extraction mode 3). The color code is the same as used in Figure 1. Different RdhA proteins are shown with different line types.

Extraction mode 3 was characterized by cell lysis via beat beating in the presence of detergent at room temperature (~20°C) (Kublik et al., 2016). Although some foam formed and a part of the proteins was expected to be lost with it, it is a very fast and direct procedure and the BN-PAGE analysis could be started 20 min after addition of the detergent. After BN-PAGE the gel was cut into 64 gel slices that were analyzed for their protein content by mass spectrometry. In total 116 proteins were identified. Among these 116 proteins again all OHR complex subunits were identified (Table 2). Out of the 32 encoded RdhA paralogs five were identified, CbdbA80, CbdbA84, CbdbA1588, CbdbA1618, and CbdbA1638. As with the two other extraction modes, only one out of the 32 encoded RdhB, CbdbB3, was identified. The OHR complex disintegrated to an intermediate degree into modules but the modules were intact. The relative protein abundance of the OHR complex subunits across the blue native gel lane showed organization in at least three modules (Figure 4). One such module consisted of the subunits OmeA/OmeB/HupX and had its maximum relative protein abundance in slice 20, corresponding to a size of ~540 kDa. Interestingly, a trace peak of HupL was also found in slice 20. A second smaller maximum for OmeA and HupX was found in slices 34 and 35, however, OmeB distribution was different in these slices. The second module formed by HupL and HupS showed very similar relative protein abundance distribution patterns over all slices for those two proteins. The maximum was found in slice 49 at a size of ~60 kDa. A smaller part of this module was also found together with the maximum of RdhA CbdbA80 and RdhB CbdbB3 in slice 40 at ~135 kDa. RdhA CbdbA80 and RdhB CbdbB3 showed the same maximum in slice 40 and also a similar overall distribution patterns thus forming a third module. As mentioned above, this module seemed to interact weakly with the HupL/HupS module. Although also other RdhA proteins were identified in slice 40 in minor amounts they had their maxima either at higher molecular mass (CbdbA1638 ~250 kDa, CbdbA84 and CbdbA1588 ~242 kDa) or for RdhA CbdbA1618 at a lower molecular mass of ~75 kDa. This showed separation of different RdhA along the blue native gel lane. A remarkable observation is the occurrence of secondary maxima of OmeB and RdhB CbdbB3 in slice 32 independent of OmeA and HupX to which OmeB is normally tightly bound. No other RdhB proteins than CbdbB3 were identified in the gel. Therefore, it is not possible to judge if co-localization of these RdhB proteins with their respective RdhA occurred.

**Figure 5:**
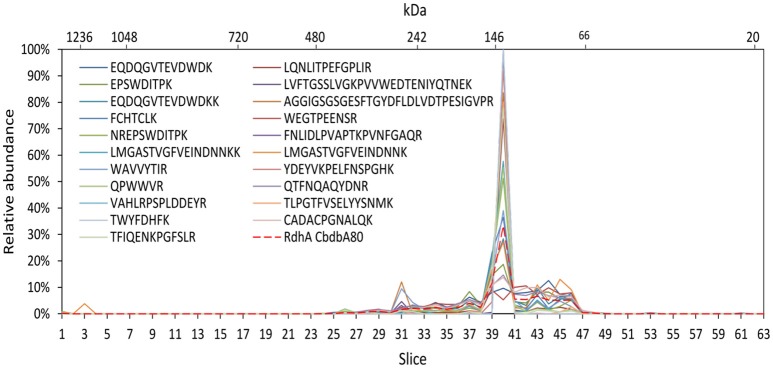
Analysis of single peptide distributions across the blue native gel lane. Relative distribution of RdhA CbdbA80 across the gel lane (red dashed line) together with the distribution of all single detected peptides of RdhA CbdbA80 after protein extraction according to mode 3. The peptide maxima are all in slice 40.

To test the reliability of our method we compared the distribution of OHR complex proteins across the gel lane with the distribution of each peptide detected of the specific protein because this distribution should be the same for each peptide. Indeed, this behavior was seen in all OHR complex proteins as exemplified in Figure 5 for the RdhA CbdbA80. However, this calculation also showed that the distribution patterns became much more stable and better resolved when more peptides in a protein were detected. In this way, the distribution of proteins with less identified peptides (for example RdhB CbdbB3) are expected to be less reliable than proteins with many identified peptides. Analyzing the peptide distribution for all OHR complex subunits it was observed that besides of the expected situation that all peptides share the maximum of relative peptide abundance with the maximum of relative protein abundance of their respective protein, also a distribution was observed that the relative peptide abundance was split into two or more maxima in slices next to each other (Supplemental Figures 1, 2). OHR subunits were peptides showed such a split of maxima for relative peptide abundance were OmeA, RdhA CbdbA84 (Supplemental Figure 1C), and RdhA CbdbA1588 (data not shown).

### Hierarchical cluster analyses

**Figure 6:**
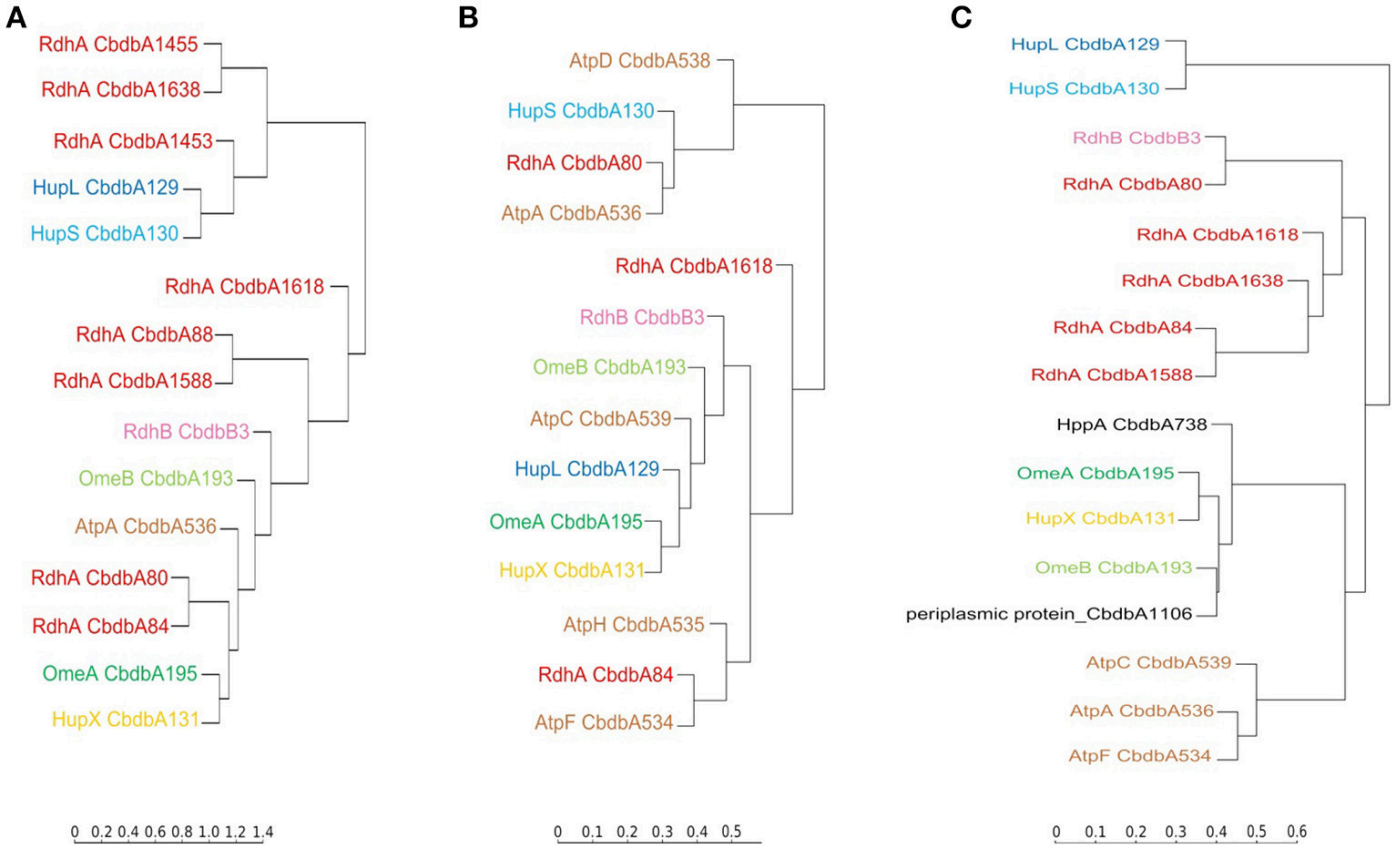
Cluster analysis of the distribution profiles of proteins suspected to be part of the OHR complex across blue native gels after application of the three extraction modes. **(A)** Distribution on blue native gels after extraction mode 1; **(B)** after extraction mode 2; **(C)** after extraction mode 3. The cluster analysis places proteins with similar migration pattern together. Colors are used as elsewhere in this study. ATP synthase subunits (brown) are added as reference. Two other proteins having a similar distribution of relative protein abundance across the gel in extraction mode 3 as the OmeA/OmeB/HupX module are displayed in black. The full analyses are given in Supplemental Figure 3.

Hierarchical cluster analyses comparing distribution patterns of the different proteins across gel slices were calculated for all three extraction modes to quantitatively describe interactions between subunits (Figure 6 for proteins suspected to be within the OHR complex; Supplemental Figure 3 for all detected proteins). As suggested by the distribution graphs (Figures 2–4), the trees confirm that extraction mode 3 preserved the integrity of the three modules HupL/HupS, OmeA/OmeB/HupX, and RdhA/RdhB CbdbB3 and at the same time separated them from each other and from other protein complexes such as the ATPase complex (Figure 6C). HupL and HupS were clustering closely together and clearly formed a cluster distinct from other OHR complex proteins and other proteins (Supplemental Figure 3C). Also OmeA/OmeB/HupX formed a cluster of proteins with very similar slice distribution. It is worth noticing, that HupX followed even closer the gel distribution of OmeA than the membrane protein OmeB. Two further proteins were identified to cluster with the OmeA/OmeB/HupX module, which are annotated as a hypothetical periplasmic protein (CbdbA1106) and a K^+^-insensitive pyrophosphate energized proton pump HppA (CbdbA738) (Figure 6C). Similar to HupS and HupX, CbdbA1106 is predicted to be located on the outer side of the cytoplasmic membrane with the largest part of its 254 amino acid residues, anchored to the membrane by one single transmembrane helix at the N-terminus. In contrast, CbdbA738 is predicted to be a large 708 amino acids containing integral membrane protein with 15 transmembrane helices. Although the cluster analysis cannot differentiate between specific and unspecific interactions it gives evidence for a very similar distribution across the native gel as seen for the OmeA/OmeB/HupX module. In the cluster analysis of the complexome analysis RdhA proteins were separated from the main peaks of the other suspected OHR complex proteins. RdhA CbdbA80 clustered directly with CbdbB3, the RdhB protein associated with it in the genome. Also RdhA CbdbA84 and RdhA CbdbA1588 were very similarly distributed in the gel slices and formed a distinct subcluster in the cluster analysis, however, in the phylogenetic tree of RdhA proteins they are not particularly closely related to each other (Hug et al., 2013).

Extraction mode 2 in contrast led to almost complete disruption of all modules into their subunits (Figure 6B). This can best be seen at the ATPase subunits distributed across the tree. Even the otherwise tightly bound HupL/HupS module was disrupted. Interactions that can still be observed under these strongly dissociating conditions were the association of OmeA with HupX and the association of OmeB with RdhB CbdbB3, as tentatively observed in Figure 3. These two interactions seem to be the strongest in the OHR complex of strain CBDB1.

Extraction mode 1 (Figure 6A) more strongly conserved the integrity of the OHR complex than extraction mode 2 (almost complete disruption of the complex and modules) and 3 (disruption of the complex but preservation of module integrity). Therefore, interactions of modules can be monitored under extraction mode 1. The strong interactions between OmeA and HupX and between OmeB and RdhB are conserved. Also the HupL/HupS interaction is maintained indicating that this interaction is also strong even if less than the previously mentioned interactions. Also weaker interaction were preserved: between the hydrogenase module (HupL/HupS) and the RdhA proteins CbdbA1455, CbdbA1638, and/or CbdbA1454; and between CbdbA80 and CbdbB3.

The cluster analysis reveals the presence of further complexes within the proteome of strain CBDB1 (Supplemental Figure 3C). For example, the three subunits of the ATP synthase complex detected in complexome analysis 3, AtpA, AtpC and AtpF, corresponding to the alpha (F_1_ unit), epsilon (F_1_ unit) and b subunit (F_0_ unit), shared very similar distribution profiles of relative protein abundance across the gel slices. All three had their maximum relative abundance in slice14, corresponding to a molecular weight of about 780 kDa. In the cluster analysis AtpA and AtpF clustered closer together than each with AtpC. Another identified protein complex in the analysis of extraction mode 3 was the cytoplasmatic Vhu hydrogenase complex with its two subunits VhuA and VhuG closely clustering in their distribution profile. The same was found for CarA and CarB the small and large subunit of the carbamoyl-phosphate-synthase. From the riboflavin biosynthesis pathway RibE (riboflavin synthase)/RibH (6,7-dimethyl-8-ribityllumazine synthase) were identified which do not cluster together but share their maximum of relative protein abundance in slice 13. For PurQ/PurL the phosphoribosylformylglycinamide synthase I and II of the purine biosynthetic pathway (maximum in slice 36) of complexome analysis 3 the same observation was made. More proteins involved in the purine biosynthetic pathway were detected, PurA, PurD, PurE and PurH, but did not cluster together and also did not share the maximum in relative protein abundance indicating the absence of a larger protein complex. Furthermore, a module of the pyruvic-ferredoxin oxidoreductase was identified. The subunits PorA and PorB clustered together although they had their maxima in relative protein abundance in slice 32 and 33 of complexome analysis 3, respectively. The third subunit PorG had its maximum in relative protein abundance also in slice 32, but was in the cluster analysis slightly distant from the other two subunits. The last subunit PorD was not identified in this analysis. None of the 11 subunits of the predicted complex I (Nuo) was identified in the gel slices indicating it was at low abundance. Only two of the 53 encoded ribosomal subunits were identified in complexome analysis 3 RplA (L1) and RplL (L7/L12), both 50S ribosomal proteins, and they also were present in one cluster.

## Discussion

In this study we provide detailed information on a large protein complex in *D. mccartyi* strain CBDB1 able to catalyze a “stand-alone” respiration, dependent only on one protein assembly, not on quinoid electron mediators. To our knowledge, this is the only known example yet of such an autonomous respiration directly with physiological electron donors and electron acceptors within one protein complex. Although few other bacteria (acetogens) and archaea (methanogens) have been described to lack quinones (Schoepp-Cothenet et al., 2013), they still generate a proton or sodium gradient across the membrane by oxidizing and reducing mediating cofactors, such as ferredoxin or NADH, not the substrates directly. Therefore, it is important to study this OHR complex in more detail even if the applicable methods are strongly confined to those with very good sensitivity, excluding the majority of standard biochemical characterization procedures. We here explored successfully the potential of complexome analyses for the characterization of the OHR complex. By using different protein extraction modes, representing different solubilization strengths, we also obtained information about the dynamics of the protein interactions. In addition we acquired evidence for the presence of at least three modules in the complex and evidence on specific protein-protein interactions within the complex.

Complexome analyses can give a detailed description of cellular complexes (Wittig et al., 2006; Wittig and Schägger, 2009). In combination with high-resolution mass spectrometry a high protein identification depth can be reached allowing to trace many proteins in one single experiment. In our study, we applied only low amounts of proteins onto the native gels and obtained good resolution of protein clusters but no strong detection depth, meaning that we only detected the most abundant proteins. Indeed, in comparison to previous work, describing the full proteome of *D. mccartyi* strain CBDB1 (Schiffmann et al., 2014) the total number of detected proteins in the present study is low. However, we aimed at good resolution of protein subcomplexes in the gel and less on the identification depth because we focused on the composition of the abundant OHR complex and protein-protein interactions within this complex. We also obtained experimental evidence for this argumentation when we got much better spatial focusing of RdhA proteins when they were at low concentration than when they were at high concentration (CbdbA88, CbdbA1453, CbdbA1588 and CbdbA1638 with narrow focus vs. CbdbA80 and CbdbA84 with broader distribution Figure 2). In all three complexome analyses done in this study, all seven suspected OHR complex subunits were detected and especially in the complexome analysis 3 OHR modules were well separated from each other but still intact. In addition to the information on the complex composition these results again confirm the dominant role of the OHR complex in the physiology, biochemistry and evolution of *Dehalococcoides* species. Another reason impacting the total number of detected proteins in our analyses was the choice to optimize the instrument measurement time toward more precursor scans (MS1 scans) than done in previous studies (Schiffmann et al., 2014; Kublik et al., 2016) to obtain more precise label-free quantification results.

Complexome analysis data depend strongly on the protein extraction mode, as also exemplified by our data. With different detergent types, detergent concentrations, temperatures and incubation times different solubilization strengths can be applied resulting in different observable protein-protein interactions. Each of the three complexome analyses presented here is a snapshot of one particular solubilization degree. In our previous work we have evaluated different detergents (Kublik et al., 2016) but here we focused on DDM at a concentration of 1% and varied the incubation time and preparation sequence in the different extraction modes. Although we started with the largest amount of cells in complexome analysis 2 (using extraction mode 2 which included membrane preparation by ultracentrifugation) the resulting complexome data indicates that only a low amount of protein was applied to the gel, meaning that a large portion of protein was lost during the ultracentrifugation step. This is indicated by the total amount of area counts for all detected proteins together (1.9E + 10), which was only half of that found after extraction mode 1 (3.9E + 10) (Table 2) although 16 times more cells were used for the complexome analysis 2 (calculated to the applied volume onto the gel). Also the relative distribution of the proteins and modules on the gel showed that extraction mode 2 described for *Desulfobacula toluolica* (Wöhlbrand et al., 2016) was not conserving OHR complex integrity (Figure 3). All together this indicates that membrane isolation by ultracentrifugation is heavily biasing the results leading to massive loss of proteins. It is surprising that many ribosomal proteins were detected in the membrane preparations (extraction mode 2) indicating that ribosomes were enriched by this procedure. The extraction mode 2 however, was useful in observing tight interactions between integrated membrane proteins which seem also to be enriched with this mode. For the analysis of loosely attached peripheral membrane proteins the extraction modes 1 and 3 were better suited, especially because much less protein was lost. Still, in extraction mode 3 much more protein was lost than in mode 1 as seen by the total area counts after mass spectrometry (Table 2). This might be due to foam formation when cell lysis was done in the presence of detergent. However, complexome analysis 3 provided a precise separation of the OHR complex modules.

From the distribution of single peptide identifications all stemming from the same protein, confidence for the results could be drawn. Overall, the peptide distributions were very reliable and matched the protein distribution, indicating that false positive detections did not play a major role. Generally that also means that the obtained results were more stable for proteins with more peptide identifications than for those with few or only one peptide identification, most notably RdhB CbdbB3 and RdhA CbdbA1618. Exceptions, where not all peptides showed the exact same distributions were RdhA CbdbA84, RdhA CbdbA1588 and OmeA, where peptide relative abundance maxima split into two maxima in neighboring gel slices (Supplemental Figure 1). We cannot explain this observed behavior, but we speculate that interactions with other proteins partially prevented the tryptic digest in some slices and therefore changed the relative abundances.

One of the major challenges of this study was the solubilization and mass spectrometric detection of integral membrane proteins. The OHR complex is believed to contain two integral membrane proteins, OmeB (10 predicted transmembrane helices) and RdhB (three predicted transmembrane helices). Only three OmeB peptides and only one single peptide from the 32 RdhB proteins encoded in the genome were identified in all our mass spectrometric data together. The single peptide identified from RdhB CbdbB3 had the sequence FIQYLK and is located in the cytoplasmic loop of the protein, requiring two trypsin cuts on the cytoplasmic sides of two transmembrane helices. This is only possible if the loop is not interacting with other proteins. Accordingly, the detection of this peptide across the whole blue native lane supports that the RdhA is bound to the extracytoplasmic surface of RdhB as considered in our model (Figure 1). The peptide also allowed the tracing of interactions with its associated RdhA CbdbA80, but we were not able to monitor other RdhB proteins. These difficulties in detecting integral membrane proteins are often described and are due to problems in solubilization, trypsin cleavage and fragmentation. This is especially true for RdhB proteins which are very small proteins with only about 90 amino acids and have only few trypsin digestion sites of which some might be protected by protein-protein interactions. The detection of further RdhB proteins by mass spectrometry is a topic that needs to be addressed in the future.

With extraction mode 3 three different OHR complex modules were observed, mostly separated from each other. Each of the modules contained several single protein subunits which interacted more strongly with each other than the modules interacted with each other. The largest module consisted of OmeA, OmeB and HupX with a relative protein abundance maximum of about 540 kDa. HupX showed a much stronger interaction with OmeA than with the other two hydrogenase subunits HupL and HupS, although it is encoded in an operon with HupL/HupS. This result is in agreement with previous results showing co-localization of HupX with OmeA/OmeB in *Dehalococcoides* strains (Kublik et al., 2016; Hartwig et al., 2017) but we can here confirm this with better spatial resolution and also by a clustering calculation. However, this higher resolution also indicated that the calculated size of the module (181.6 kDa) is not fitting to the size where the relative protein abundance maximum was identified (~540 kDa). Consequently this module is either interacting with other proteins or polymerizes to a homotrimer. Potential further interaction partners of the OmeA/OmeB/HupX module where identified by cluster analysis (Figure 6C) where we found identical migration patterns for the hypothetical periplasmic protein CbdbA1106 and a K^+^ insensitive pyrophosphate energized proton pump CbdbA738/ HppA. Interestingly CbdbA1106 and HppA were identified also in previous analyses on BN-PAGE and SDS-PAGE of surface cross-linked samples of strain CBDB1, but not on 2D BN/SDS-PAGE (Kublik et al., 2016). Due to the similar predicted topology of CbdbA1106, HupS and HupX observed interactions could also be unspecific via interactions of the transmembrane helices. HppA is thought to utilize the energy from pyrophosphate hydrolysis to generate a proton motif force and it is predicted to have 15 transmembrane helices. Until now it is not known how protons are translocated across the membrane by the OHR complex. One hypothesis describes OmeB as potential proton pump with a glutamate residue in helix eight possibly involved in proton pumping (Zinder, 2016). Another possibility is that an additional protein such as HppA is interacting with the complex to generate the proton motif force needed for ATP synthesis.

The second module identified in extraction mode 3 consisted of the Hup subunits HupL and HupS with a maximum of relative protein abundance at a molecular weight of ~60 kDa. The calculated size of HupL and HupS together is 95 kDa and is therefore higher than indicated by BN-PAGE. However, this seems to be a typical migration behavior of smaller protein assemblies in BN-PAGE analyses and is influenced by detergents and Coomassie G-250 additive (Crichton et al., 2013). The separation of HupL/HupS from the other complex subunits in BN-PAGE analyses after solubilization was found previously (Kublik et al., 2016).

In contrast to previous approaches we could separate clearly different RdhA module versions by BN-PAGE. Whereas, complexome analysis 1 did not separate the two most abundant RdhA proteins CbdbdA80 and CbdbA84, they were well separated in complexome analysis 3. The reason, why CbdbA84 was at a much higher molecular mass than CbdbA80 is not clear. However, we know that CbdbA84 is an active reductive dehalogenase (Adrian et al., 2007b). A similar separation between two RdhA proteins can be seen with CbdbA1588 (together with CbdbA84 at 240 kDa) and CbdbA1638 (together with CbdbA80 at 140 kDa). Interestingly, the two single separated peaks of CbdbA1588 and CbdbA1638 can also be seen in complexome analysis 1, suggesting that the separation was only efficient for RdhA proteins at low concentration. Never, RdhA proteins were found at the monomeric mass of ~55 kDa. Taking the results from complexome analyses 1 and 3 together it appears that the Rdh module at 240 kDa was attached to the OmeA/OmeB/HupX module whereas the Rdh-module at 140 kDa was associated with the HupL/HupS module. A separation of RdhA proteins into bands between 130 and 300 kDa was seen before in 2D BN-PAGE/SDS-PAGE gels and was dependent on the detergent type concentration (Kublik et al., 2016). We here show that the complexes with different sizes contain a different complement of RdhA proteins. Only by observing this separation between RdhA proteins we could for the first time observe the co-migration of an RdhA protein with the RdhB protein located with it in the genome. This strongly suggests that indeed there is a specific interaction between an RdhA and an RdhB protein. It has to be investigated if this is transferrable to other RdhA/RdhB interactions.

Previously the main reductive dehalogenase activity in BN-PAGE gels was identified at a position of around 242 kDa with 1,2,3,4-tetrachlorobenzene as electron acceptor (Kublik et al., 2016). This correlates well with position of the RdhA CbdbA84 identified here in our complexome analysis 3. CbdbA84 has previously been described as chlorobenzene reductive dehalogenase (CbrA) (Adrian et al., 2007b).

Our results also clearly indicate module-module interactions that can be seen in the two module-conserving analyses 1 and 3. In analysis 3 the different modules were mostly separated from each other. However, minor peaks, where a small part of one module putatively interacted with another module, were located at different positions than the main peak. This can be observed in Figure 4 where the main part of the HupL/HupS module was located in slices 47–51, whereas a minor part interacted with the RdhA/RdhB module in slices 39–41. This is the same peak that was observed in complexome analysis 1 (Figure 2) in slice 20 as shown by the indicative presence of the single peak of the RdhA CbdbA1453. In this slice 20, however, almost all of the HupL/HupS module was present whereas only a minor fraction of the HupL/HupS module was migrating together without RdhA subunits to slice 24. This shows that the solubilization strength in complexome analysis 3 was higher than in complexome analysis 1 and that therefore in complexome analysis 1 still a major part of the HupL/HupS module was associated with the RdhA protein(s). A tiny but significant portion of the HupL/HupS module was also co-migrating with the OmeA/OmeB/HupX module in slice 20 of complexome analysis 3 (Figure 4). Another module-module interactions can be seen in complexome analysis 1 (Figure 2) between the OmeA/OmeB/HupX module and the RdhA/RdhB module in slice 18.

Taking the results of our current study together we might need to revise the hypothesis of a linear model for the OHR complex in which HupL/HupS, OmeA/OmeB/HupX and RdhA/RdhB are linearly organized (Kublik et al., 2016). Especially, because we here found evidence for the direct interaction of HupL/HupS with RdhA/RdhB without mediation by OmeA/OmeB/HupX in slice 40 of complexome analysis 3 (Figure 4) an in slice 20 of complexome analysis 1 (Figure 2). Hence, we now prefer a triangular model in which all three modules are interacting with each other. However, we still do not have evidence for the stoichiometry between the different modules in the complex and also we do not have information about the number of subunits in each of the three modules. It could be expected that such a stoichiometry can be inferred from the size estimation of the blue native gels using the applied marker, but we have realized that this size estimation is not precise and complexes often migrate different from what is expected. For example the HupL/HupS module always migrates at about 60 kDa although the two proteins together should already have 95 kDa. Also the OmeA/OmeB/HupX module together with a theoretical mass of 180 kDa was located in gel slices corresponding to a molecular weight of around 540 kDa. The RdhA-containing modules are always distributed within the range between 130 and 260 kDa and it would be very speculative to predict the subunit composition of the submodules. A more precise size estimation is necessary and therefore, the elucidation of the exact stoichiometry and organization of the complex needs to await further work.

In addition to information on the OHR complex we also obtained evidence for several other protein-protein interactions in strain CBDB1. However, the absence of any further highly expressed protein complexes is more surprising than the detection of expected interactions between subunits such as VhuA/VhuG or AtpA/AtpF/AtpC. Indeed most remarkable is the absence of indications for a complex I (Nuo) which is encoded in the genome with all necessary subunits with the exception of the NADH-input domain NuoEFG.

## Author contributions

KS and LA conceived the study and designed the experiments. KS and JK did the lab experiments, KS, JK, and LA analyzed the data KS, JK, and LA wrote the manuscript.

### Conflict of interest statement

The authors declare that the research was conducted in the absence of any commercial or financial relationships that could be construed as a potential conflict of interest.

## Supplementary material

Supplemental Figure 1 Analysis of single peptide distributions across the native gel lane together with the respective proteins. **(A)** HupL, **(B)** OmeA, **(C)** RdhA CbdbA84. Splitting of peak maxima was observed in OmeA and CbdbA84.

Supplemental Figure 2 Hierarchical cluster analysis of the distribution across a blue native gel for all peptides of the OHR complex after extraction mode 3. The analysis indicates that peptides originating from the same protein mostly cluster together confirming the stability of our analyses. The three peptides that are isolated on the top were detected only once in a single slice each, explaining their separation in the tree. The color code is the same as elsewhere in this study, with exception of RdhA proteins. RdhA proteins are colored as following: CbdbA80, red, CbdbA84, purple, CbdbA1588, black, CbdbA1618, dark red, and CbdbA1638, bright red.

Supplemental Figure 3 Cluster analysis of all detected proteins in the three complexome analyses. **(A)** after extraction mode 1; **(B)** extraction mode 2; **(C)** extraction mode 3. OHR-complex proteins and ATPase subunits are colorized as elsewhere in this study.

Supplemental Table 1 List of all detected proteins and their identified peptides. The list gives absolute numbers of area counts determined by label-free quantification. The numbers represent the sum of all area count values obtained from the gel slices. Also the number of peptides identified for each protein is listed.

Supplemental Table 2 Color code for the suspected OHR complex proteins used in this manuscript.

## Abbreviations

DDM: N-dodecyl-ß-D-maltoside
BN-PAGE: blue native polyacrylamide gel electrophoresis
OHR: Organohalide respiration.
