# Supplementary figures and images for: The Complexome of Dehalococcoides mccartyi Reveals Its Organohalide Respiration-Complex Is Modular
Source: Front Microbiol. 2018 Jun 12;9:1130. doi: 10.3389/fmicb.2018.01130 (PMC6005880; doi:10.3389/fmicb.2018.01130)

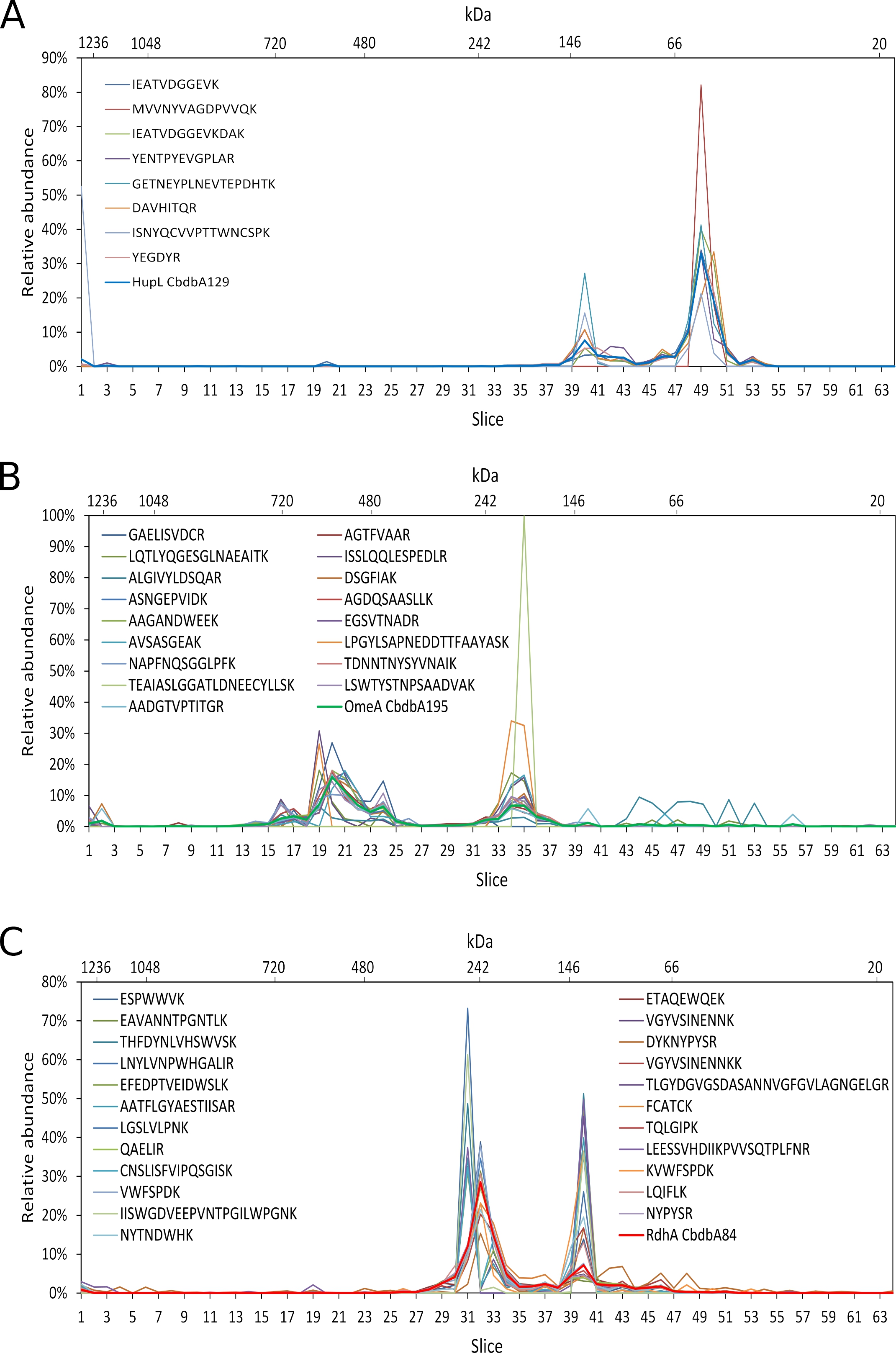

Supplement: Supplemental Figure 1 — Analysis of single peptide distributions across the native gel lane together with the respective proteins. (A) HupL, (B) OmeA, (C) RdhA CbdbA84. Splitting of peak maxima was observed in OmeA and CbdbA84. [file Image_1.JPEG]

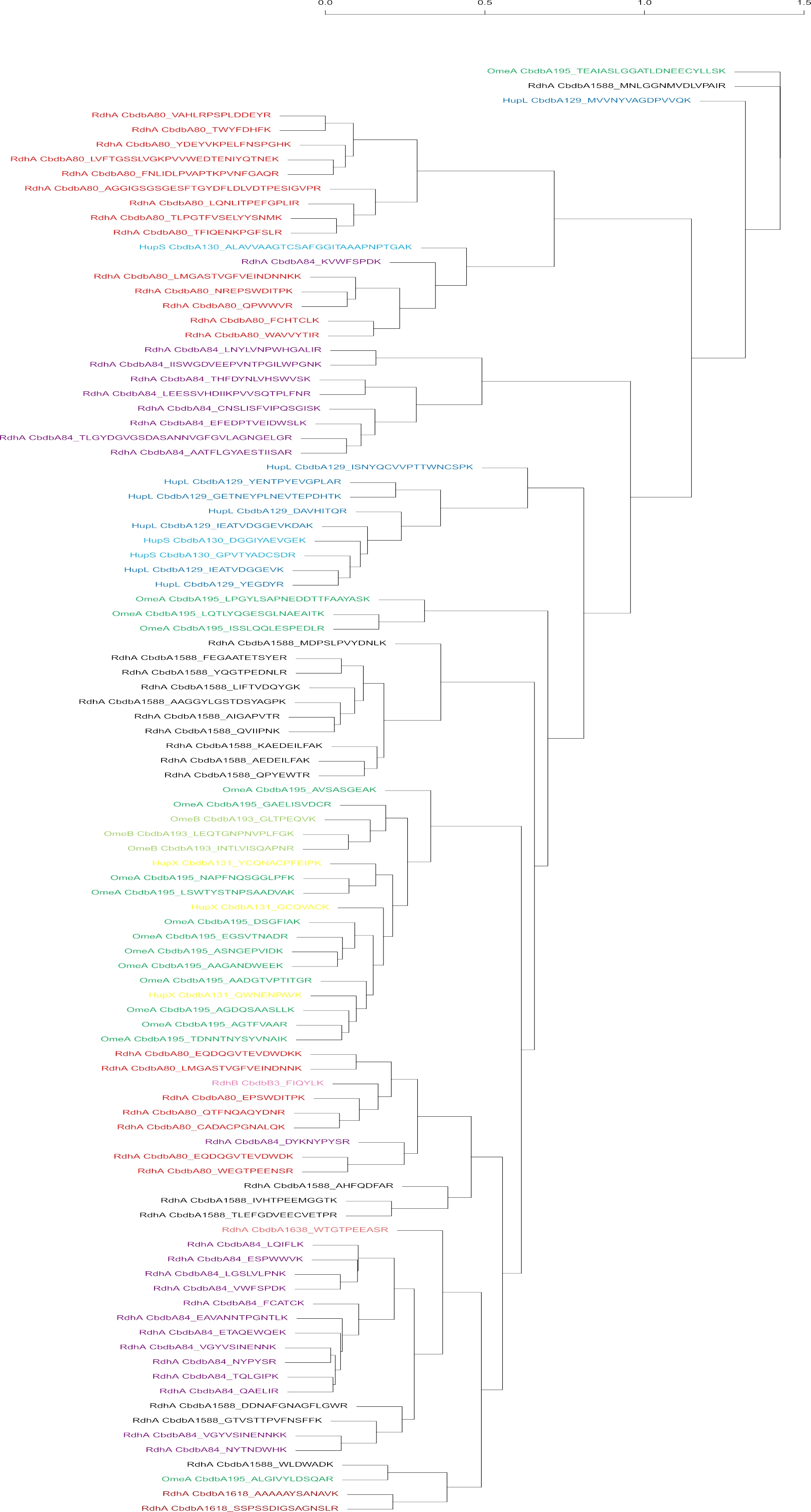

Supplement: Supplemental Figure 2 — Hierarchical cluster analysis of the distribution across a blue native gel for all peptides of the OHR complex after extraction mode 3. The analysis indicates that peptides originating from the same protein mostly cluster together confirming the stability of our analyses. The three peptides that are isolated on the top were detected only once in a single slice each, explaining their separation in the tree. The color code is the same as elsewhere in this study, with exception of RdhA proteins. RdhA proteins are colored as following: CbdbA80, red, CbdbA84, purple, CbdbA1588, black, CbdbA1618, dark red, and CbdbA1638, bright red. [file Image_2.JPEG]

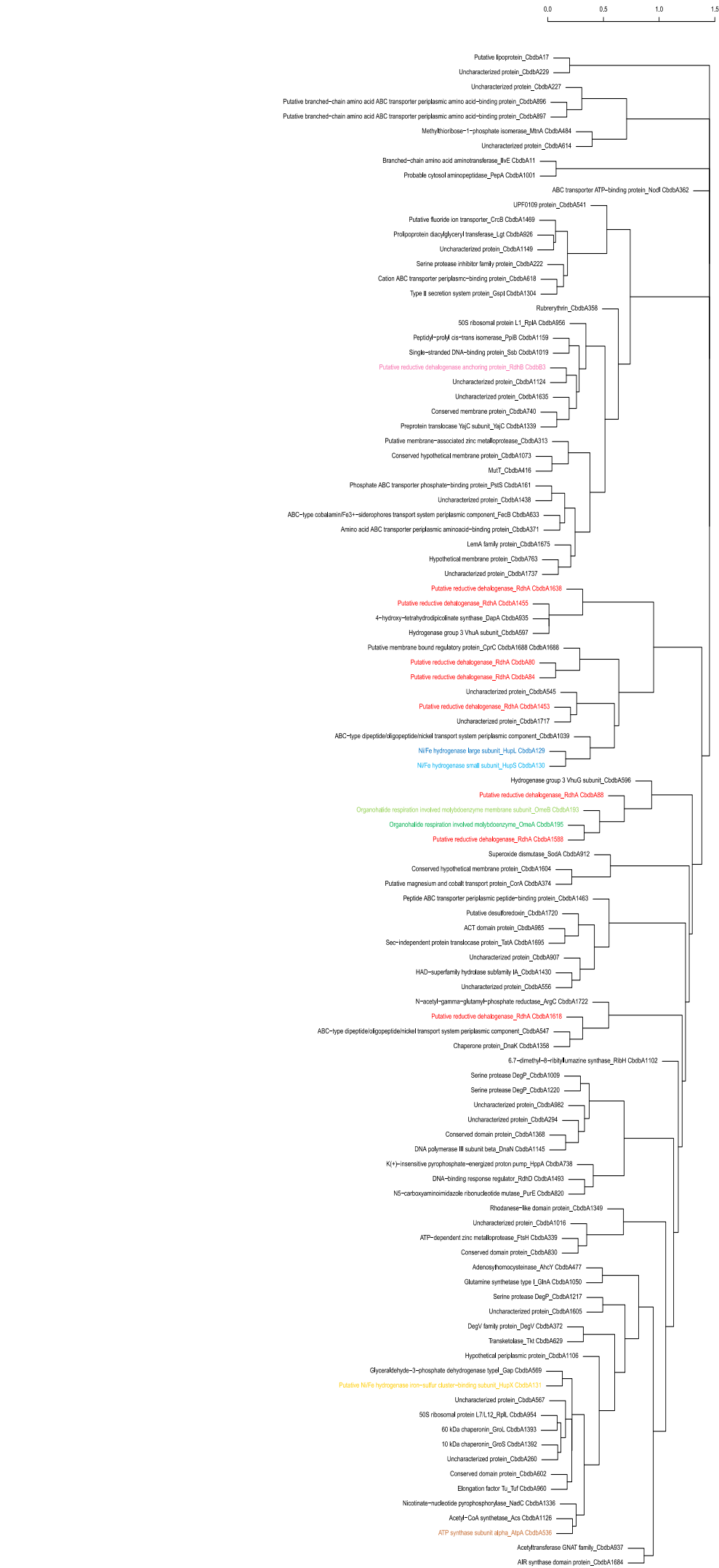

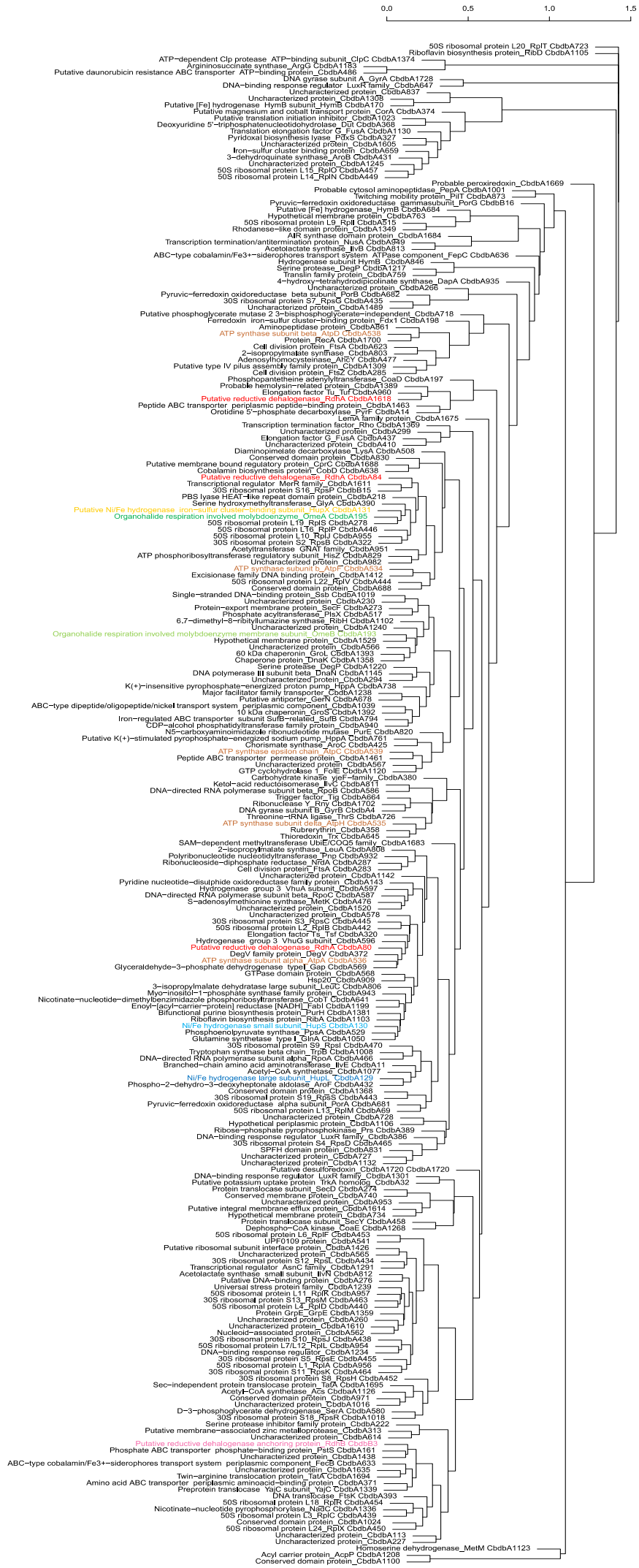

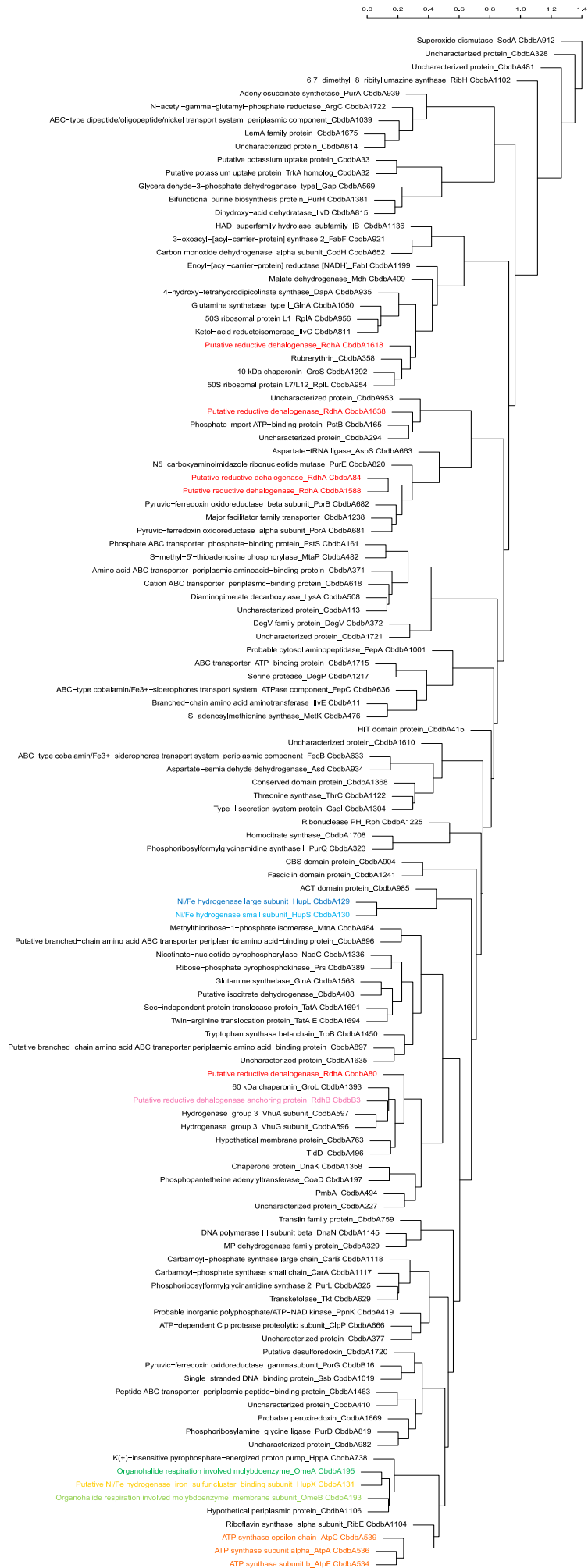

Supplement: Supplemental Figure 3 — Cluster analysis of all detected proteins in the three complexome analyses. (A) after extraction mode 1; (B) extraction mode 2; (C) extraction mode 3. OHR-complex proteins and ATPase subunits are colorized as elsewhere in this study. [file Image_3.PDF]
